# New approaches to high-resolution mapping of marine vertical structures

**DOI:** 10.1038/s41598-017-09382-z

**Published:** 2017-08-21

**Authors:** Katleen Robert, Veerle A. I. Huvenne, Aggeliki Georgiopoulou, Daniel O. B. Jones, Leigh Marsh, Gareth D. O. Carter, Leo Chaumillon

**Affiliations:** 1 National Oceanography Centre, University of Southampton Waterfront Campus, European Way, Southampton, SO14 3ZH United Kingdom; 2 School of Earth Sciences, University College Dublin, Belfield, Dublin, D04 V1W8 Ireland; 3 Earth Institute, University College Dublin, Dublin, Belfield, Ireland; 4 Ocean and Earth Science, University of Southampton, European Way, Southampton, SO14 3ZH United Kingdom; 5 Marine Geoscience, British Geological Survey, The Lyell Centre, Research Avenue South, Edinburgh, EH14 4AP United Kingdom; 6 L’Institut national des sciences et techniques de la mer (INTECHMER), Boulevard de Collignon, Tourlaville, Cherbourg, 50110 France

## Abstract

Vertical walls in marine environments can harbour high biodiversity and provide natural protection from bottom-trawling activities. However, traditional mapping techniques are usually restricted to down-looking approaches which cannot adequately replicate their 3D structure. We combined sideways-looking multibeam echosounder (MBES) data from an AUV, forward-looking MBES data from ROVs and ROV-acquired videos to examine walls from Rockall Bank and Whittard Canyon, Northeast Atlantic. High-resolution 3D point clouds were extracted from each sonar dataset and structure from motion photogrammetry (SfM) was applied to recreate 3D representations of video transects along the walls. With these reconstructions, it was possible to interact with extensive sections of video footage and precisely position individuals. Terrain variables were derived on scales comparable to those experienced by megabenthic individuals. These were used to show differences in environmental conditions between observed and background locations as well as explain spatial patterns in ecological characteristics. In addition, since the SfM 3D reconstructions retained colours, they were employed to separate and quantify live coral colonies versus dead framework. The combination of these new technologies allows us, for the first time, to map the physical 3D structure of previously inaccessible habitats and demonstrates the complexity and importance of vertical structures.

## Introduction

Habitat structural complexity may have a profound effect on ecological interactions^1^. Availability of different physical structures allows for more microhabitat types, greater niche space, and can increase biological diversity^2, 3^. For a given scale, certain distinct spatial structures (termed ‘keystone structures’) may be particularly crucial in providing the resources necessary to support a large number of species^4^. Within marine environments, vertical structures may play such a role by providing access to hard substrate for attachment of sessile species and by potentially creating more complex hydrodynamic patterns^5, 6^. For example in the deep sea, vertical structures can host a variety of species assemblages^7, 8^ as well as particularly high abundances of certain species of cold-water corals^9–11^. In turn, structural complexity created by these cold-water corals may provide associated organisms with higher attachment points, potentially improving access to food particles, additional living space, or offer protection from predation^12^. As vertical structures form a natural protection against the impacts of trawling, and have the potential to be an important source of larvae for recolonization of previously damaged habitats^7^, understanding the ecology of these ecosystems is of considerable interest.

The study of assemblages on vertical structures, however, had previously been impaired by technologies, with traditional sampling methods such as cores, trawls and/or towed video systems being inappropriate^13^. The traditional downward-looking approach to mapping using ship-board multibeam echosounders (MBES), particularly in deeper waters, smoothens rugged topography and fails to image under overhanging cliffs. In order to solve these issues, a new technique for the forward mapping of vertical structures was introduced, whereby a MBES was mounted on the front of a Remotely Operated Vehicle (ROV) and survey lines were carried out by moving the vehicle sideways along the wall^7^. As a result, much higher resolution maps and estimates of coral framework cover could be achieved using this novel technique. The separation of live coral colonies from dead coral framework however, was not possible and relied on accompanying video transects.

Stereo-camera systems are increasingly being used for three dimensional (3D) reconstructions of shallow complex marine habitats, such as tropical coral reefs^14–16^. Closely related photogrammetry techniques such as ‘Structure from Motion’ (SfM), an approach that allows 3D models to be reconstructed from overlapping images of a single moving camera, are also being employed to build high-resolution digital elevation terrains of such reefs^17–19^ and representations of single coral colonies at such fine resolutions that individual polyps can be reconstructed^20^. From these techniques, high-resolution coloured point clouds are produced, georeferenced, scaled, and used for morphometric measurements as well as to derive fine-scale terrain metrics.

Although both ROV forward mapping and SfM photogrammetry techniques can provide very high resolutions, they remain limited in the spatial extent they can cover. As a result, Autonomous Underwater Vehicles (AUVs) are routinely used to cover larger seafloor areas^21^. Although AUVs can provide higher resolutions than ship-mounted systems in many deep-water environmental settings (e.g. abyssal hills^22^, cold-water coral and rocky reefs^21, 23, 24^ or manganese nodule fields^25, 26^, the AUV-mounted MBES had mainly remained restricted to top-down acquisition, therefore failing to resolve vertical structures that may be present in more complex habitats.

In this study, we make use of three novel mapping techniques to reconstruct ecologically important vertical habitats in 3D. We employ (1) sideways-looking AUV MBES, (2) front-mounted ROV MBES and (3) SfM photogrammetric reconstructions of ROV video transects. With these environments mapped at finer resolutions than previously achievable, we are able to derive very fine-scale terrain metrics and precisely position individual organisms in 3D space. This allows us to (1) investigate the spatial positioning of *Acesta excavata* clams and reef-building cold-water corals (mainly *Lophelia pertusa*, *Madrepora oculata* and *Solenosmilia variabilis*) and (2) explain spatial variation in ecological characteristics (such as abundance, number of species observed and diversity) of megabenthic invertebrates.

## Methods

### Survey Description

**Figure 1:**
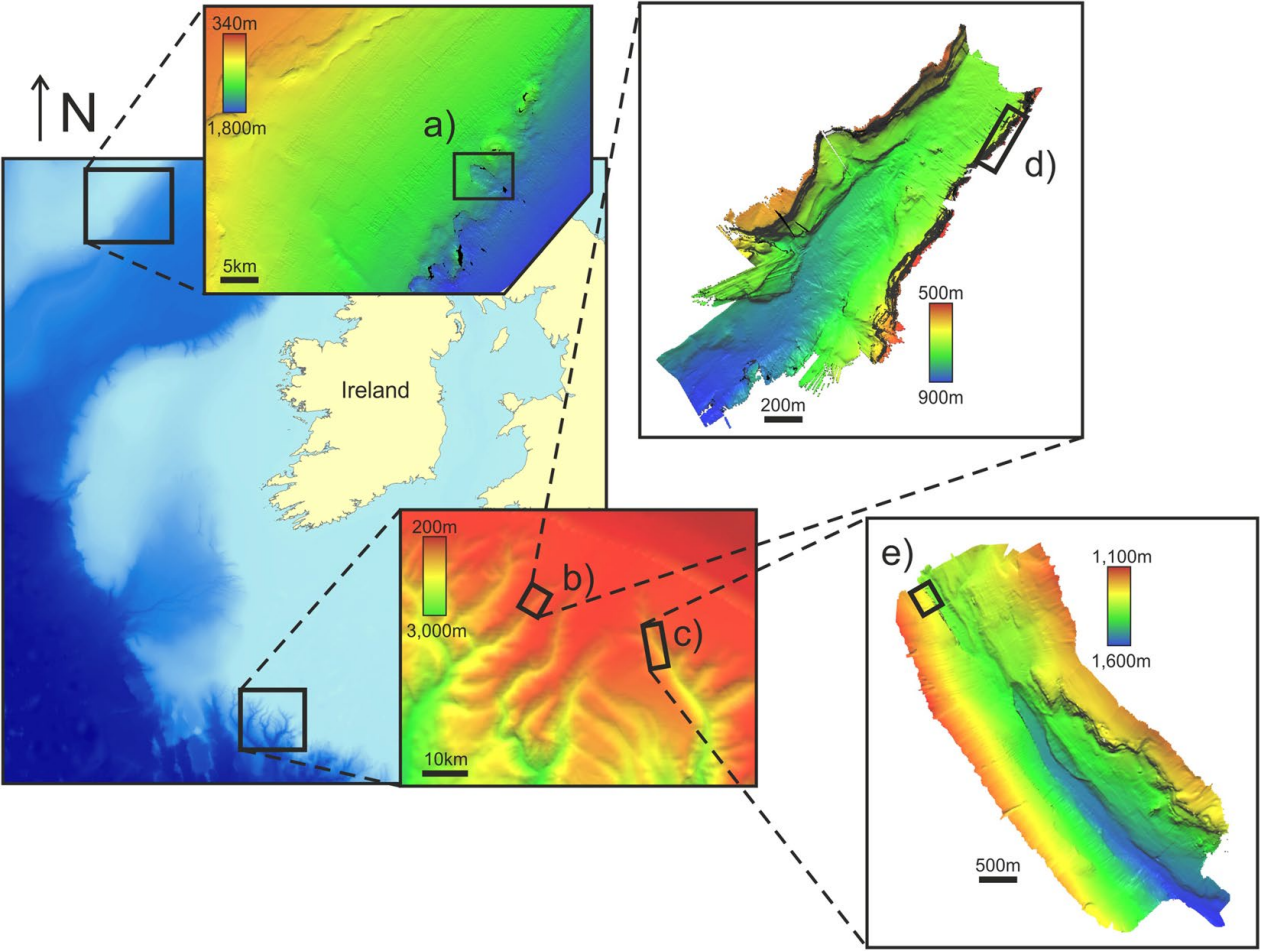
Maps showing the location of the survey datasets. (**a**) Rockall Escarpment, (**b**,**c**) Whittard Canyon AUV sideways mapping and location of the ROV vertical mapping of (**d**) the Acesta Wall and (**e**) the Coral Wall. No AUV bathymetry was available for the Rockall Escarpment area. General background bathymetry from the General Bathymetric Chart of the Oceans (GEBCO http://www.gebco.net/) and region specific bathymetries from INFOMAR (http://www.infomar.ie/). General map created in ESRI ArcMap (version 10.2.2) and site specific maps created in CloudCompare (version 2.6.1).

Three walls were mapped and imaged at high resolutions over the course of three research cruises. In July 2014, onboard the RV *Celtic Explorer* (cruise CE14011) and in September 2015, onboard the RRS *James Cook* (cruise JC125), vertical structures were mapped using front-mounted ROV multibeam (Reson Seabat 7125, 512 beams) and video cameras, on the Rockall Escarpment and in the Whittard Canyon respectively (Fig. 1). The CE14011 cruise used the ROV *Holland I* as part of the study on ‘Slope Collapses on Rockall Bank and Escarpment Habitats’ (SORBEH) while the ROV *Isis* was on board JC125 and was part of the CODEMAP project (‘Complex Deep-sea Environments: Mapping habitat heterogeneity As Proxy for biodiversity’). The wall mapped during SORBEH represented part of a headwall scarp within the Rockall Bank Slide Complex^27^ while the one mapped in Whittard Canyon during JC125 had first been discovered by Johnson *et al*.^8^. The former faced south and started at a depth of ~1530 m, extending upward for ~110 m with a gradient of 50–60° while the later was ~200 m in height, faced northwest with a nearly vertical slope, starting from a depth of ~740 m. During a previous cruise in 2009 (JC-36), a northeast facing ~120 m high wall (maximum depth of 1400 m, gradient of ~70°) in the eastern branch of Whittard Canyon had been mapped using *Isis* (Huvenne *et al*. 2011). The AUV *Autosub6000*, a 6000 m depth-rated vehicle developed at the National Oceanography Centre in Southampton^28^, was also used to map the two branches (Mission 94 and Mission 97) where the Whittard Canyon walls were located. Table 1 summarizes the cruises, vehicles and dives for each wall.

**Table 1:** List of ROV dives and AUV missions carried out during surveys of three walls.

Wall	ROV	Cruise (Imagery)	Imagery	Cruise (ROV MBES)	ROV (MBES)	Res (m)	AUV	Cruise (AUV MBES)	AUV (MBES)	Res (m)
Rockall Escarpment	*Holland I*	*Celtic Explorer* 14011	Dive 10	*Celtic Explorer* 14011	Dive 11	0.4	NA	NA	NA	NA
Whittard Canyon, Coral	*Isis*	*James Cook* 125	Dive 249	*James Cook* 036	Dive 123	0.5	Autosub 6000	*James Cook* 125	Mission 97	5
Whittard Canyon, Acesta	*Isis*	*James Cook* 125	Dive 255	*James Cook* 125	Dive 264	0.4	Autosub 6000	*James Cook* 125	Mission 94	0.5

MBES: multibeam echosounder, Imagery: video transects converted to Structure from Motion.

### Multibeam Mapping

**Figure 2:**
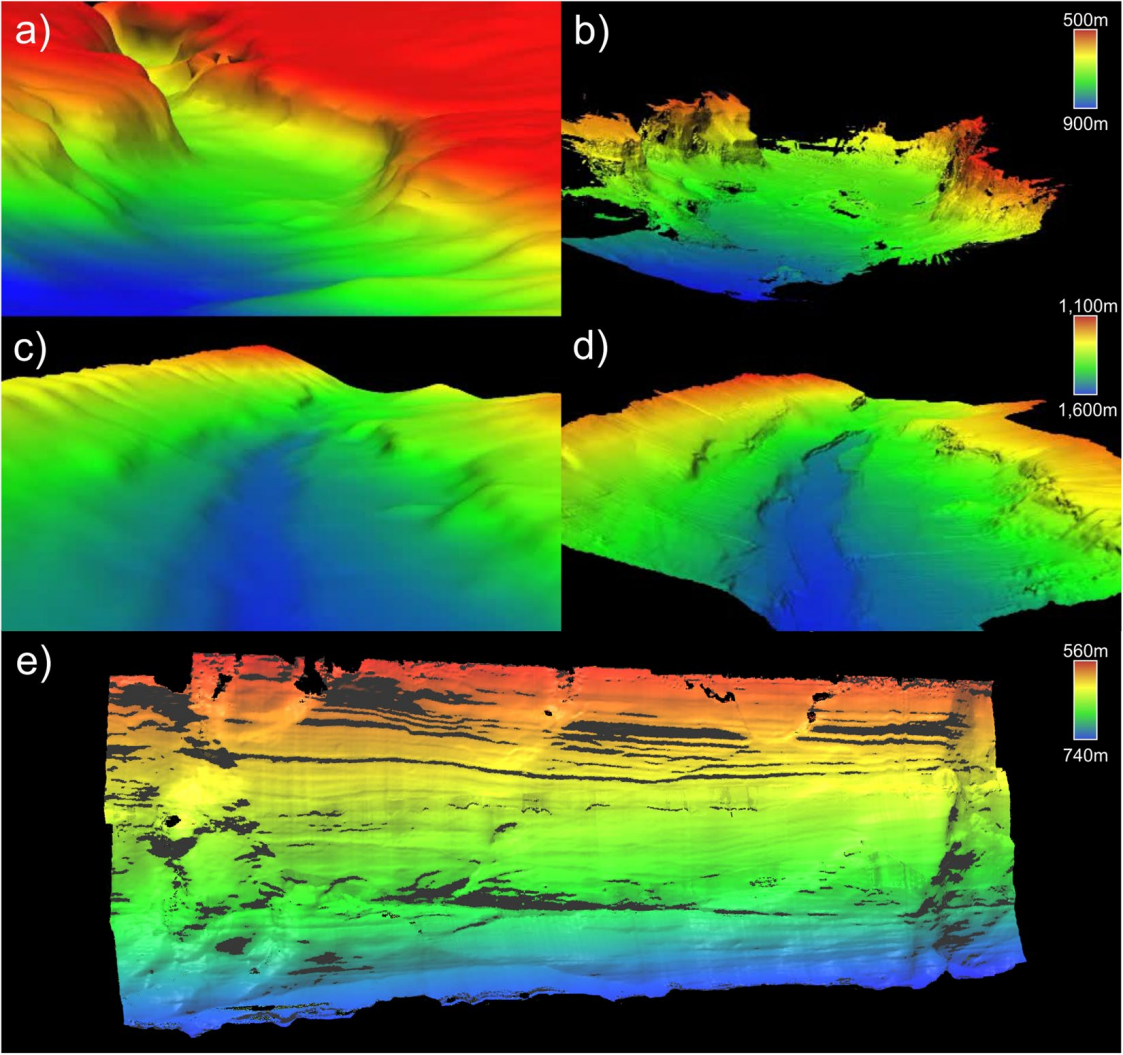
Spatial reconstructions obtained using each acoustic approach. Point clouds were transformed into meshes for display using a Poisson surface reconstruction (in CloudCompare) (**a**,**c**) ship-board EM120 downward-looking multibeam echosounder (MBES) (resolution 50 m), (**b**,**d**) sideways-looking AUV mounted MBES (**b**) resolution 0.5 m; (**d**) resolution 5 m and (**e**) ROV front-mounted MBES (resolution 0.4 m). Whittard Canyon Acesta Wall (**a**,**b**,**e**) and Whittard Canyon Coral Wall (**c**,**d**). A fly through video is available as Supplementary Information S1 and a 3D pdf of the AUV bathymetry is available as Supplementary Information S2–3.

The techniques developed for MBES forward mapping of walls using ROVs have been described in detail in Huvenne *et al*.^7^ (JC036 Dive 123) and Huvenne *et al*.^29^ (CE14011 Dive 11). In brief, as applies to JC125 Dive 264, the following data processing steps were carried out. The multibeam data were collected with a Reson 7125 MBES operated at 400 kHz, were converted from .pds to .s7k format using PDS2000 (version 3.7) and were imported into the CARAIBES software (IFREMER). When possible, Doppler navigation was employed, but when Doppler lock could not be achieved, smoothed ‘Ultra-Short-Base-Line-System’ USBL data (Sonardyne systems) were used instead. Navigation and vehicle depth data were extracted to text files and subjected to a coordinate transformation in the statistical computing software R (version 3.2.3, The R Foundation for Statistical Computing, 2015); this was done to project the vertical wall horizontally for further processing. The vehicle attitudes were then transformed to represent the new configuration and applied; with the original pitch becoming the new roll, the original roll becoming the new heading offset, the new pitch having been calculated by inverting the sign of the original heading deviation from the direction of survey, and the new vehicle depth as obtained from the coordinate rotation. The horizontally projected data could then be edited, gridded and exported to. xyz files. The .xyz files were then imported into R and a directional cosine filter was applied to remove remaining corrugation artefacts resulting from noise in the USBL data. Finally the data were back rotated to a vertical position and projected to UTM zone 29 (Whittard Canyon) or 28 (Rockall Escarpment) and exported as.xyz for importation as 3D point clouds into the software CloudCompare (version 2.6.1, Fig. 2e) for terrain analysis.

*Autosub6000* was equipped with an EM2040 multibeam system, which was employed at 200 kHz for Mission 97 and 400 kHz for Mission 94, with the doubling of the number of beams enabled. For better coverage of vertical structures, the system was mounted to look sideways with a 20° roll offset from the traditionally down-looking position. All missions were processed in the CARAIBES software (Fig. 2b,d).

### Photogrammetry

**Figure 3:**
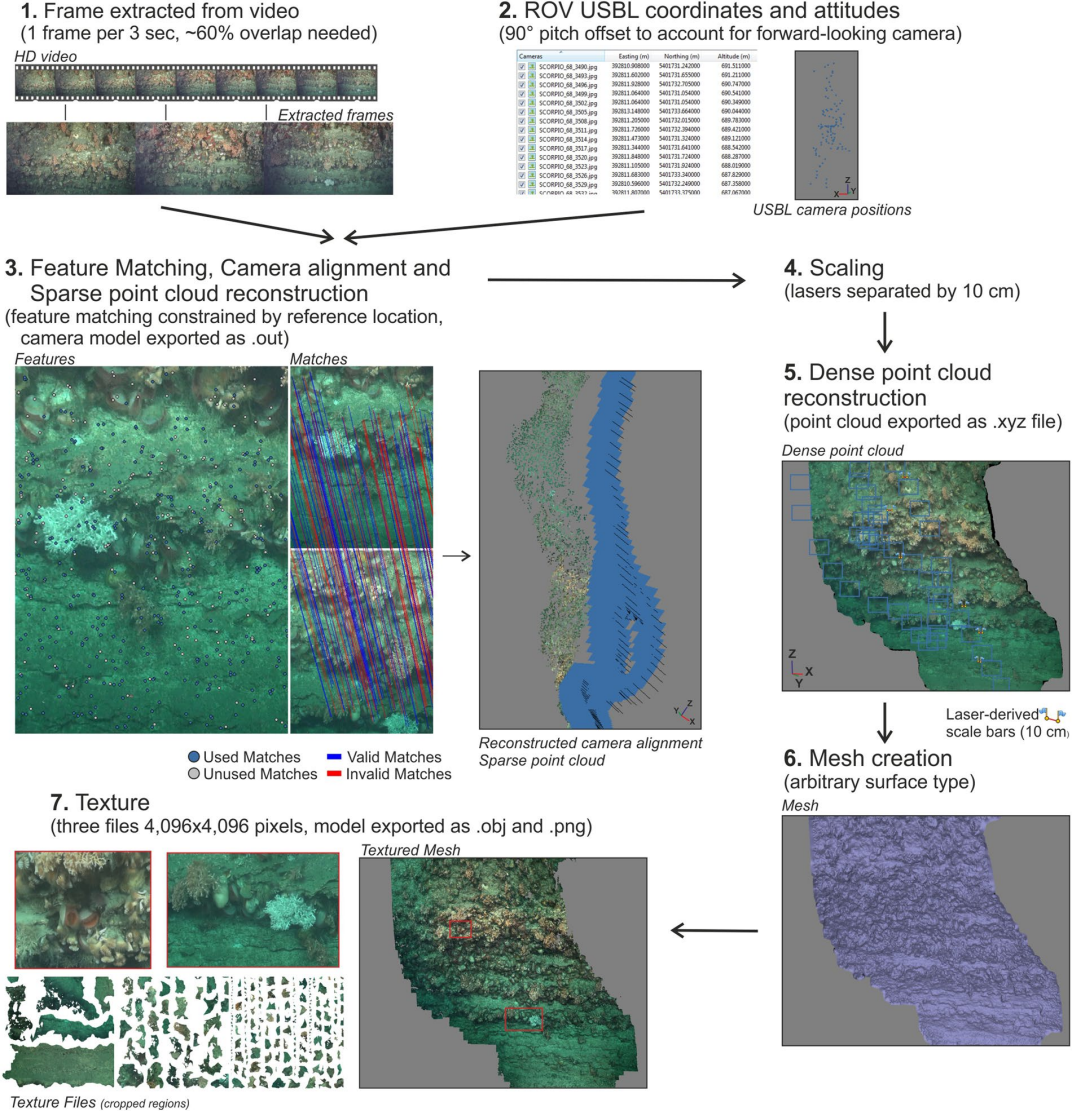
Flowchart of the steps taken to created 3D reconstructions from the ROV video.

For each of the walls mapped, an additional ROV dive was carried out with the aim of filming the megabenthos. The ROV *Holland I* was equipped with an OE 14366 Colour Zoom Camera while an Insite Super Scorpio was installed on the ROV *Isis* (both 1920 × 1080 pixels). The software Agisoft PhotoScan professional edition (v1.2.4) was employed to build 3D image reconstructions using a technique called ‘Structure from Motion’ (SfM)^30^. In deeper waters, this technique has been successfully applied to examine geological features from ROV video surveys^31–33^, but its application for ecological surveys so far remained limited (but see ref. 34). A detailed description of the approach as applies to this study is provided as Supplementary Information S4 and is summarized in Fig. 3.

**Figure 4:**
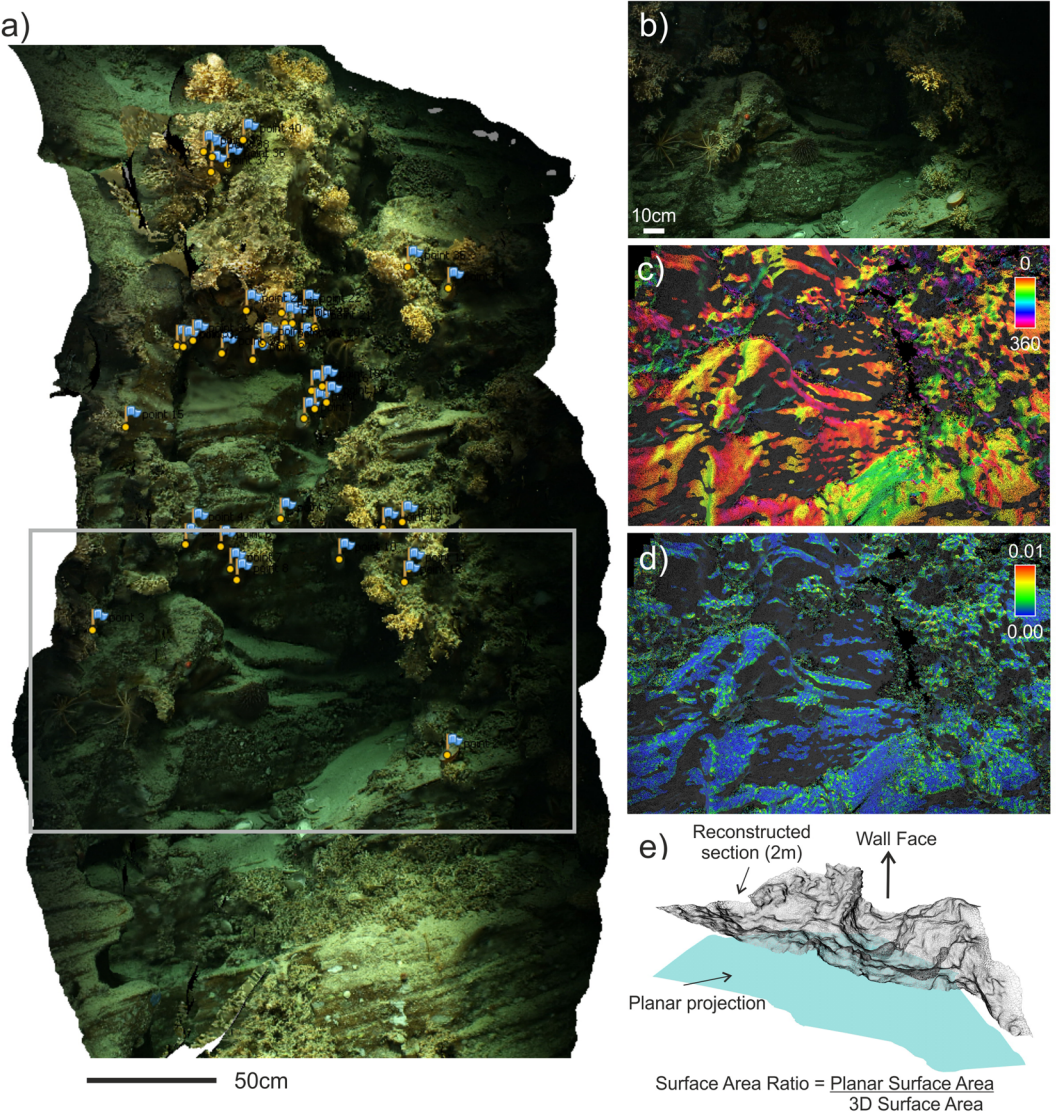
Structure from Motion reconstruction and derived terrain variables. (**a**) 3D reconstruction (textured mesh) from images taken along the Rockall Escarpment. Marker flags show the emplacement of *Acesta* clams and the grey box delineates the outline of the extracted frame shown in (**b**). Derived terrain variables: (**c**) aspect calculated at 0.05 m, (**d**) roughness at 0.02 m and (**e**) rotated view of a reconstructed section (2 m) of wall showing how surface area ratio was calculated, the grey mesh shows the 3D reconstruction while the blue plane shows the 2D area. An animated video is available as Supplementary Information S5 and a 3D pdf is available as Supplementary Information S6.

The 3D reconstructions were used to examine the fine-scale spatial positioning of the main dominant species (mainly *Acesta excavata* clams, and cold-water corals: *Lophelia pertusa*, *Madrepora oculata* and *Solenosmilia variabilis*). A marker was set in Agisoft Photoscan to precisely georeference each individual within the point cloud (Fig. 4). Markers were exported as .xyz text files for the statistical analyses. An additional benefit of this step is that once a marker is positioned in a frame, it also appears in all other frames covering the same position. This greatly minimizes the risk associated with double counting individuals if the ROV crossed over previously covered terrain. For each individual organism, terrain variables (refer to next section) were assigned by computing the mean of the 10 nearest points.

In addition, for a more extensive section of the Coral Wall (~500 m^2^), which was comprised of overlapping horizontal survey lines, manual classification was conducted to estimate the percentage cover of live coral and dead framework. This was carried out in Agisoft PhotoScan by manually delineating areas within the dense point cloud which represented coral colonies (identified based on colour and overall shape) and assigning them to their respective class.

### Terrain Analysis

The point cloud processing software CloudCompare was used to compute terrain descriptors. The normals for each. xyz text file were recomputed using a height function with radii of 0.02, 0.05 and 0.1 m (to produce a terrain analysis at multiple scales) and were then converted to dip/dip direction to obtain slope and aspect values (Fig. 4c,d) (later converted into eastness and northness in R). Gaussian roughness for each scale was also calculated and the resulting point cloud was exported as an ascii text file for importation into R. An estimate of surface-area ratio^35^ was obtained by calculating the 3D surface area and dividing by the area of a projected plane through each 2 m section (Fig. 4e).

CloudCompare was also employed to calculate terrain descriptors at multiple scales from the MBES point clouds. For both AUV and ROV data, normals were calculated at 5, 10, 25 and 50 m, and converted to slope and aspect (also converted into eastness and northness in R) while neighbourhoods of the same size were employed to calculate gaussian roughness. Additional terrain descriptors, topographic position index (TPI), topographic rugosity index (TRI), roughness (maximum minus minimum) (see Wilson *et al*.^36^ for description of terrain descriptors) and entropy were also calculated for the same scales in R using all points within the given spherical radius (e.g, 5, 10, 25 and 50 m).

### Video Analysis

The overlapping frames extracted for the photogrammetry were imported as an ‘image sequence’ into the freely available image processing software ImageJ (National Institute of Health, version 1.6.0_20) and the ‘cell counter’ plugin was used to mark the position and taxa for each individual. All individuals larger than 20 mm were counted and identified to morphospecies when species level identification could not be achieved. Biological variables such as abundance, number of species and diversity (reciprocal of Simpson’s index, 1/D, Simpson^37^) were derived for each 2 m section. For each of these sections, averages for each point cloud derived terrain descriptor were calculated. Visibility along transects was qualitatively recorded (good, medium and poor) and sections with poor visibility were removed from the analysis. The width of each frame was estimated using parallel lasers beams projected by the ROVs for scaling purposes (100mm apart) and included as a covariate in the analyses to reduce the effect of a changing field of view.

### Statistical Analysis

‘Generalized Additive Models’ (GAMs) were used to determine which derived terrain variables were most useful in explaining observed spatial patterns in abundance, number of species and diversity (1/D). Models were built for each mapping technique (e.g. SfM, ROV MBES and AUV MBES) separately and variation partitioning^38^ was used to examine whether the inclusion of terrain variables from a different technique would provide additional information. Abundance was log + 1 transformed prior to the analysis. Variable selection was carried out by forward selection based on the adjusted R^2^, but only variables with less than 0.5 correlation (Pearson’s correlation coefficient) to other variables already present in the model were added. The value of each added environmental variable was assessed using Aikaike Information Criterion (AIC). Estimates for the adjusted R^2^ and the mean square error were derived using k-fold cross validation (e.g. dataset split into folds, and in turn, each fold is removed from the training dataset and used as the testing dataset)^39^. Such an approach helps ensure that the models are not fitted too closely to the data. Initial results showed that too few samples were available from the Coral Wall to correctly estimate smooth terms using GAMs, as such, simpler ‘Generalized Linear Models’ (GLMs) were employed.

All statistical analyses were carried out in R using the libraries ‘vegan’ and ‘mgcv’, while data manipulation made use of functions in the ‘gtools’, ‘RANN’, ‘zoo’, ‘ff’ and ‘ffbase’ packages. The last two being particularly useful for the handling of the very large SfM point cloud datasets as they allow large data structures to be stored on disk, but handled as if they were held in memory^40^.

## Results

### Fine-scale habitat selection

**Figure 5:**
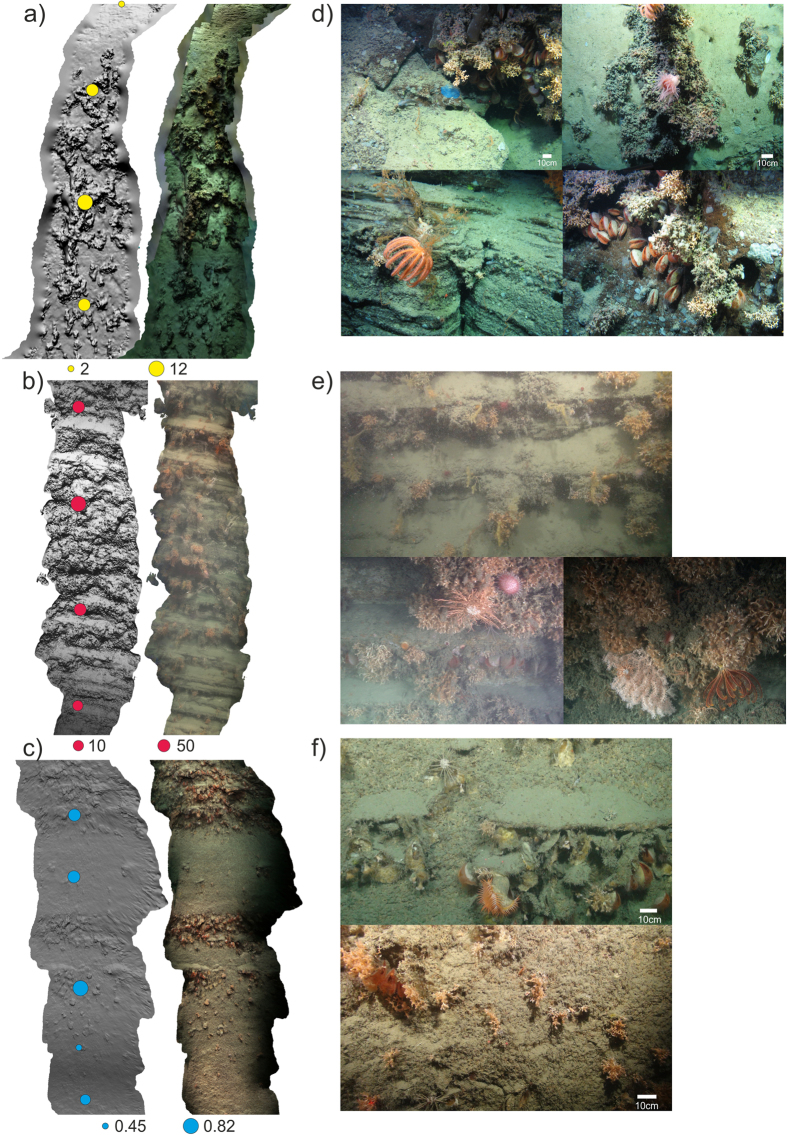
Communities observed along each wall. Examples of short video sections reconstructed using ‘Structure from Motion’ shown as 3D meshes (left), textured meshes (right) in addition to variation in ecological variables as derived from video analysis of the megabenthos (coloured dots, calculated from 2 m sections): (**a**) Rockall Wall - number of species, (**b**) Coral Wall - abundance and (**c**) Acesta Wall - diversity (1/D). Examples of frequently observed organisms and assemblages on each of the three walls: (**d**) Rockall Wall, (**e**) Coral Wall and (**f**) Acesta Wall, Whittard Canyon.

Using SfM, we were successful in recreating large portions of the acquired video footage as georeferenced 3D renditions, where individual organisms/colonies could be positioned in 3D space (Figs 4 and 5). The observed total errors in reconstructed camera locations were<10 m for location and <10° for attitude, which is in line with expected errors for USBL navigation at these depths (~1% of depth). However, these are errors relative to the bathymetry, and within each photogrammetry reconstructions, the errors associated with the positioning of individual organisms would be much smaller (scaling errors as obtained from the lasers: 1–3 mm). Errors were observed to increase towards the end of long video transects. Yaw errors tended to be the most pronounced because cameras were on a pan-and-tilt module and as a result, the vehicle heading and the camera direction were likely different.

#### Rockall Escarpment

The Rockall Escarpment Wall tended to be less steep and corals were mainly composed of *Solenosmilia variabilis* colonies. The first video transect showed that at the base of the escarpment there were volcanic rocks overlain by lithified yellowish layered sedimentary rocks of variable morphology with fractures, crevasses and caves, mainly inhabited by *Acesta* clams (Figs 4a and 5d). A second video transect, was located at the apex of the headwall scarp and started further away from the wall, where the slope was gentler (~30–40°) and comprised of light grey-coloured biogenic sedimentary layers with abundant borings. Above it, the wall became steeper (~50–60°) and yellowish sedimentary rocks were again observed until they blended with intruding volcanic rocks. A 50–60 m terrace halfway up was predominantly covered with coral rubble and some live coral, above which light grey biogenic sedimentary rocks with variable degree of borings were again encountered (Fig. 5a).

#### Whittard Canyon - Acesta Wall

The coral community on the Acesta Wall was composed of both *Madrepora oculata* and *Lophelia pertusa*, with *Acesta* clams often observed in association with small overhanging features (Fig. 5c,f). The vertical to subvertical wall generally consisted of thick to very thickly bedded (i.e. >600 mm thick beds), light orange to greyish white sedimentary units, which were favoured by coral communities. In places, these thickly bedded units were sufficiently weak to allow for boring of the surface by marine biota. Thin (60–200 mm thick) to medium (200–600 mm thick) spaced, light yellowish grey to greyish white sedimentary beds were frequently interbedded with the thickly bedded units. The *Acesta* clams were found to colonise the underside of these thinner beds, which dipped out of the cliff face to create the overhanging ledges.

#### Whittard Canyon - Coral Wall

**Figure 6:**
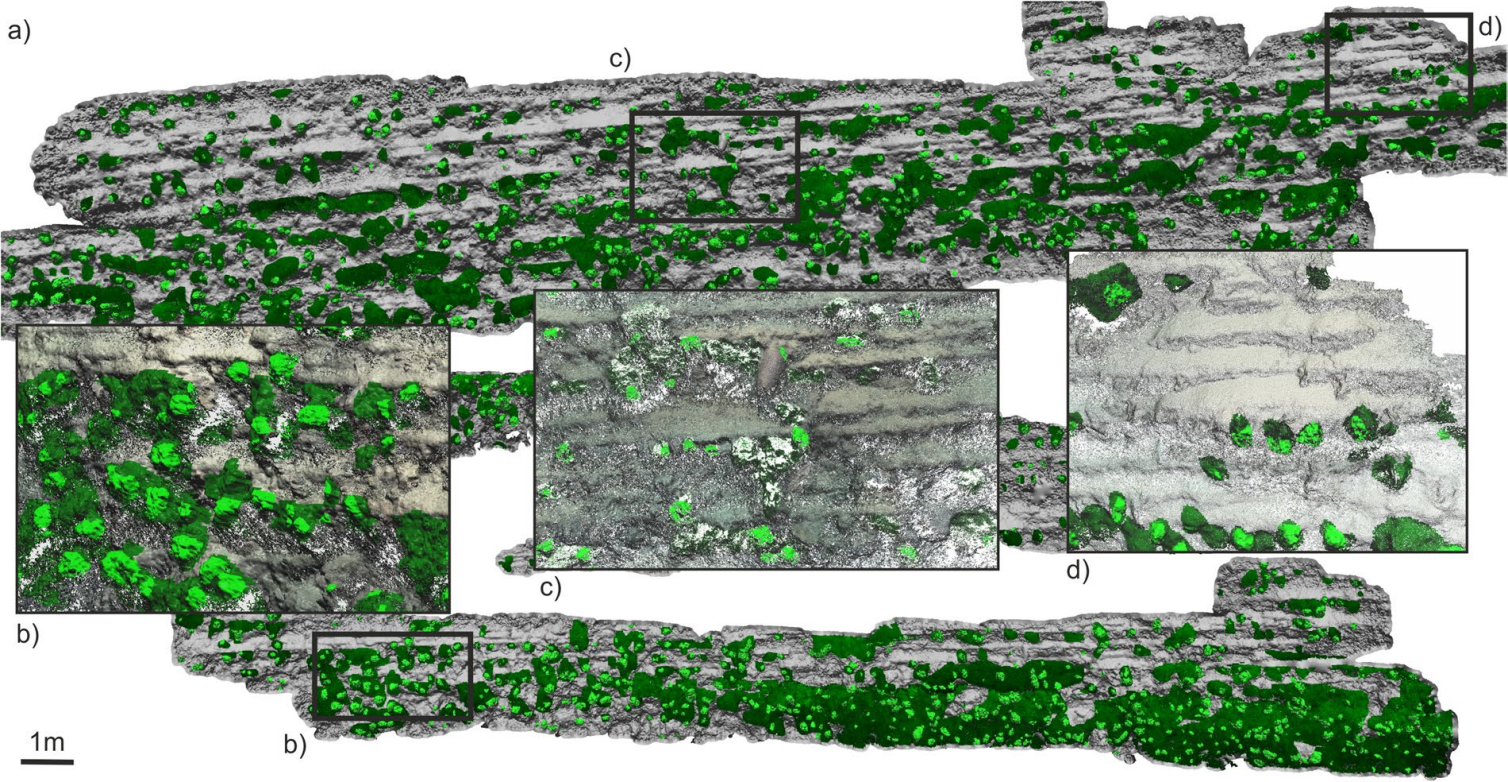
Reconstructed section of the Coral Wall. (**a**) Overview showing estimated live coral (light green) and dead coral framework (dark green) as well as zoomed in sections: (**b**–**d**).

The Coral Wall was dominated by *Lophelia pertusa*, but with some small colonies of *Solenosmilia variabilis* also being present. The lower sections of this wall were covered by such high coral densities that the associated geology could not be characterized. Visibility was also generally lower in these deeper sections. In the upper portion of this wall, alternations of strong and weak, very thin (20–60 mm thick) to thinly (60–200 mm thick) bedded sedimentary lithologies were clearly visible dipping out of the cliff face as overhanging ledges (Fig. 5e). Higher still, the strong layers were frequently very thin (20–60 mm) and often laterally discontinuous as structurally-controlled block failures resulted in the removal of sections of overhanging ledges. The slope gradient appeared to decrease in the higher section of the Coral Wall due to the reduced thickness and frequency of the stronger units. A more extensive section (~583 m^2^) was reconstructed in the shallower part (1,325–1,305 m) the Coral Wall. Manual classification of the point cloud allowed for the separation of dead coral framework (28% or ~162 m^2^) and live coral (7% or ~45 m^2^) (Fig. 6).

**Figure 7:**
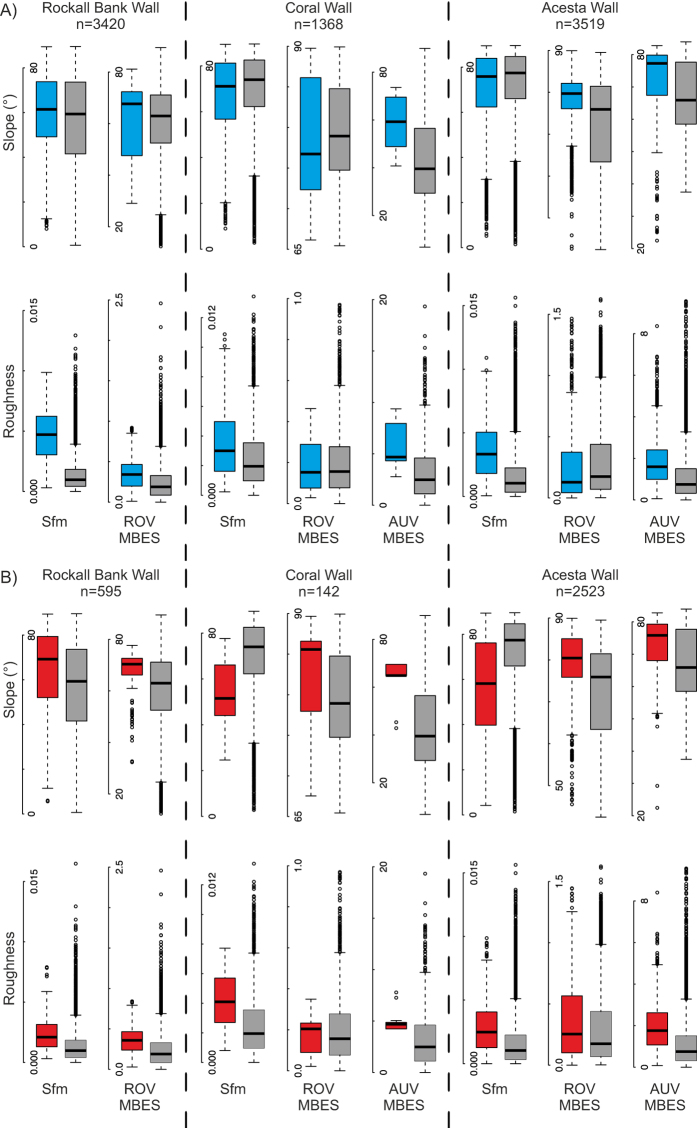
Boxplots showing the range of terrain descriptors as characterized through photogrammetry (SfM), ROV and AUV multibeam echosounder (MBES), at the locations of (**A**) cold-water coral colonies and (**B**) individual *Acesta* clams (*n*, indicates number of individuals observed). Boxes in grey show a random selection of 5000 points along the same transects.

Owing to variable image quality, identification of all individual coral colonies to species-level was not possible, but on all three walls, corals tended to be associated with higher roughness (Fig. 7). As this was particularly evident at the fine scale recorded by photogrammetry, this increased roughness was most likely due to the coral framework itself, indicating that the terrain metrics at this scale are indicative of where colonization is likely happening. However, increased roughness at coral locations was also observed using AUV MBES, which was too coarse to capture individual coral colony structure, and suggests that wall structure also has an effect. Differences in steepness between coral locations and random points were only visible in AUV derived slope, probably resulting from a tendency of the AUV to underestimate steepness of slope. On the other hand, *Acesta* clams tended to be associated with steeper slopes, except for the two Whittard Canyon walls when derived from photogrammetry. Overall these two walls were steeper than the Rockall Escarpment Wall, and *Acesta* clams on these appeared to associate with small ledges only visible in SfM reconstructions (Fig. 5c,f). Association with these small features likely explains the higher SfM-derived roughness selected, but also the apparent lower overall slopes selected at the finest scale.

### Ecological spatial patterns

Terrain descriptors derived from MBES were generally better able to explain the spatial patterns observed in abundance, number of species and diversity along the walls (Table 2). Only for abundance and diversity on the Coral Wall did SfM-derived variables result in a higher adjusted R^2^. However, much fewer samples were obtained on that wall owing to low visibility during the lower half of the video transects. The slightly coarser AUV bathymetry remained sufficient to derive terrain variables for the Acesta Wall, but point density was noticeably too sparse for the Coral Wall, resulting in no significant relationships. For abundance and number of species, the addition of finer-scale SfM-derived variables to the ROV MBES model did not improve the model. However, for diversity (1\D) of the Rockall Escarpment, the addition of the SfM-derived variables improve model fit by 13.8%, suggesting that features across a range of scales (including the structural complexity created by the coral colonies) may play an important role in driving diversity on this wall. When comparing the models for ROV and AUV MBES on the Acesta Wall, performance was not improved by combining both sets of derived variables.

**Table 2:** Environmental variables selected using Generalized Additive Models (Rockall Bank and Acesta Wall) or Generalized Linear Models (Coral Wall): Structure from Motion reconstruction (SfM), ROV and AUV multibeam echosounder (MBES). The superscripts indicate the number of samples (*n*) and folds used. TRI: Terrain Ruggedness Index, TPI: Topographic Position Index, G. Roughness: Gaussian roughness as calculated from CloudCompare, SA Ratio: Surface-Area Ratio, MSE: Mean square error.

	Site	Model	Variables Selected	R^2^	MSE
Abundance (log + 1)	Rockall Bank	SfM^1^	SA Ratio and Slope 0.05 m	0.29	0.89
		MBES ROV^1^	Slope 10 m, Roughness 50 m, Northness 50 m, TRI 05 m and G. Roughness 25 m	0.65	0.74
	Coral Wall	SfM^2^	SA Ratio, Slope 0.02 m, G. Roughness 0.02 m and Northness 0.1 m	0.77	0.19
		MBES ROV^3^	ROV TPI 05 m and G. Roughness 25 m	0.39	0.55
		MBES AUV^3^	TPI 50 m	0.23	0.44
	Acesta Wall	SfM^4^	Eastness 0.02 m, Slope 0.02 m and Northness 0.05 m	0.55	0.95
		MBES ROV^5^	Slope 10 m, G. Roughness 50 m, Entropy 25 m, Eastness 05 m and Northness 25 m	0.64	0.69
		MBES AUV^5^	Slope 25 m, Entropy 10 m, Northness 10 m and Eastness 05 m	0.63	0.84
Number of Species	Rockall Bank	SfM^1^	G. Roughness 0.05 m, Eastness 0.02 m and Slope 0.1 m	0.28	10.49
		MBES ROV^1^	Depth, Eastness 50 m, Northness 5 m and G. Roughness 05 m	0.58	6.06
	Coral Wall	SfM^2^	SA Ratio and G. Roughness 0.1 m	0.40	10.44
		MBES ROV^3^	TRI 25 m, G. Roughness 25 m and G. Roughness 10 m	0.51	8.30
		MBES AUV^3^	Not Significant	NA	NA
	Acesta Wall	SfM^4^	G. Roughness 0.05 m, Northness 0.02 m and Eastness 0.05 m	0.64	6.01
		MBES ROV^5^	Slope 25 m, Roughness 25 m, Northness 50 m, G. Roughness 10 m and Northness 10 m	0.71	4.87
		MBES AUV^5^	Slope 10 m, Eastness 10 m, Entropy 10 m and G. Roughness 25 m	0.71	4.82
Diversity (1/D)	Rockall Bank	SfM^1^	Eastness 0.1 m, SA Ratio and Northness 0.05 m	0.37	0.03
		MBES ROV^1^	TPI 50 m, Eastness 50 m, Northness 05 m and TRI 25 m	0.44	0.03
	Coral Wall	SfM^2^	Slope 0.02 m, G. Roughness 0.02 m And Eastness 0.05 m	0.51	0.02
		MBES ROV^3^	TRI 25 m, Roughness 25 m and Northness 50 m	0.44	0.05
		MBES AUV^3^	Not Significant	NA	NA
	Acesta Wall	SfM^4^	Northness 0.02 m, Slope 0.02 m and Northness 0.1 m	0.35	0.05
		MBES ROV^5^	TRI 05 m, G. Roughness 10 m and TPI 50 m	0.44	0.03
		MBES AUV^5^	Roughness 25 m, Eastness 50 m, TRI 25 m and Depth	0.47	0.03

^1^n = 94, folds = 5, ^2^n = 24, folds = 3, ^3^n = 28, folds = 3, ^4^n = 225, folds = 10, ^5^n = 175, folds = 8.

Surface-area ratio was the best predictor for abundance and number of species for the Coral Wall as well as for abundance and diversity (1\D) on the Rockall Wall. In both these cases, a higher surface area ratio (indicative of rougher terrain or presence of large coral colonies) had a positive influence. For the Acesta Wall, slope was the most important predictor variable when derived from the MBES point cloud, with positive relationships with abundance and number of species, but dropping slightly for very steep slopes when captured by the AUV. For the Rockall Escarpment, which was generally less steep than the other two walls, a more variable relationship between abundance and slope was observed, but a slight increase occurred between 50–75°. Similarly, steeper slopes, as captured via photogrammetry, were found to be associated with higher diversity for both the Coral and Acesta Walls. ROV and AUV terrain ruggedness index (TRI) also showed a positive relationship to diversity, but roughness showed more variation. Even at the very fine scales captured by photogrammetry, aspect was found to be a significant predictor, but no consistent relationships were observed. Increased fine-scale roughness on both the Rockall and Acesta Walls tended to be associated with a decrease in the number of species observed.

## Discussion

In this study, we highlight the increased level of detail and ability to map vertical and overhanging habitats in the marine environment provided by high-resolution photogrammetric reconstructions, ROV front-mounted and AUV sideways-looking MBES. We successfully apply these techniques in the deep sea to highly complex habitats and biologically diverse environments, demonstrating the importance of considering the vertical component. The ability of the derived terrain variables to describe ecological spatial patterns show applications beyond the marine environment as high resolution LiDAR or laser-scanning derived digital elevations models can also be combined with photogrammetric reconstructions.

Relationships between reef complexity and community composition have been demonstrated in shallow-water coral reefs, traditionally using the ratio of the distance covered by a metal chain laid across features on the seafloor compared to its actual length^19^. In deeper waters, as a result of such an approach being particularly challenging and the lower resolutions obtained using traditional acoustic mapping techniques, the influence of fine-scale (<1 m) habitat structures (such as coral colonies) and spatial heterogeneity on biodiversity have been more difficult to document. However, the increase in model performance observed for diversity (1/D) when combining SfM and MBES-derived terrain features highlights the likely importance of fine-scale features. These results suggest that it is not only the presence of hard substratum provided by walls that is influential, but also the complexity of habitats created by micro-features and structure building species. Our results also show that the effects of increased fine-scale complexity may differ between habitats and taxa.

The very fine roughness captured by SfM, resulting at least in part from the presence of coral framework, was found to have a positive relationship to number of species on the Coral Wall, but a negative one for the Rockall Escarpment and Acesta Walls. On the Rockall Escarpment, a large number of morphotypes were encrusting sponges mainly associated with bare rock faces while on the Acesta Wall, fewer associated species were observed growing on the smaller coral colonies. Greater numbers of species were instead seen below small overhanging features, mainly consisting of *Acesta excavata* clams, deep-sea oysters *Neopycnodonte zibrowii* and the cup-coral *Desmophyllum* sp. Conversely, on the Coral Wall, many filter-feeding species (e.g Comatulid crinoids, Alcyonacean corals, Euryalid basket stars) were observed growing on the large coral framework. In turn, the high percentage cover of coral framework may also act to reduce the number of attachment sites for other species such as *Acesta* clams, which were found in lower numbers on this wall. A detailed analysis of coral diversity across Whittard Canyon had found lower number of octocoral species associated with *Lophelia* reefs^41^, suggesting that competition between species is also responsible for structuring some of the patterns observed.

Although MBES-derived metrics were most useful in explaining ecological spatial patterns, SfM-derived metrics were particularly useful for the description of very fine-scale habitat selection as resolutions of less than 1 cm (~4–10 points per cm^2^) were achieved. Recent studies, carrying out SfM reconstructions of cold-water coral colonies under laboratory conditions were able to achieve even greater resolutions and take highly accurate morphometric measurements (e.g. surface area and volume)^20^. Bennecke *et al*.^34^ were able to carry out similar, but *in situ* analyses which allowed for coral growth rates to be estimated, highlighting the non-destructive nature of this technique. Greater spatial coverage than could be achieved using any single photograph was obtained in our study, allowing for larger structures to be visualized and analysed as a whole. Combining this with the high precision positioning of individual organisms in 3D means that we can now examine local processes acting at the level of individual organisms. In addition, important measurements (e.g. volume and 3D surface area) can now be derived and employed as quantitative indicators of habitat extent to assess ecosystem health or monitor recovery^42^. The 45 m^2^ of live coral measured on the 483 m^2^ section of Coral Wall reconstructed in this study will be able to serve as an initial dataset against which future changes can be compared. Because SfM dispenses with the complex acquisition system associated with stereo cameras, if an area had previously been filmed, it is possible to process *a posteriori* these older videos and obtain a longer time-series^34^. Software such as CloudCompare includes tools specifically designed to measure differences between two point clouds and has been successfully used to monitor cliff erosion over time on land^43^.

Distinctions between dead coral framework and live coral patches are facilitated in SfM reconstructions, which preserve colours, particularly as highly detailed textures can be overlain on meshes. For the section of the Coral Wall reconstructed in this study, coral coverage was only estimated to represent 35% of the wall (28% dead coral framework and only 7% live coral). This is in contrast to Huvenne *et al*.^7^ who estimated, through MBES mapping, a deeper section as being 70% coral-covered, but differentiation between live and dead coral was not possible. Although this same section of the wall was revisited during JC125, poor visibility prevented SfM reconstructions in these deeper sections of the Coral Wall. The few images which could still be acquired indicated that coral coverage was indeed higher in the section mapped by Huvenne *et al*.^7^.

This comparison highlights two of the main limitations of the SfM approach: the need for high visibility and the time it takes to achieve large coverage. In order to map ~500 m^2^ of a wall, over 2500 frames and nearly 3hrs of ROV video footage were required while the resulting high-resolution reconstruction took over 10hrs of computing time and the resulting dataset was over 12 Gb. These were carried out on a regular desktop computer (16 Gb of RAM, 3.4 GHz), but for larger structures, high performing computers or purpose-built desktops may be required. High resolutions could still be achieved with both the AUV and ROV MBES, and in the case of the former, poor visibility was of no consequence and greater efficiency could be achieved. The AUV mapped 2.3 km^2^ in 4 h50 in the canyon branch harbouring the Acesta Wall and 12.6 km^2^ in 13 h30 in the Coral Wall branch. This is compared to 0.032 km^2^ in 3 h30 and 0.102 km^2^ in 7 h20 using the ROV. Other scientific activities could be performed concurrently to the AUV surveys, but to complement acoustic datasets, additional time needed to be scheduled to carry out visual surveys (for both biology or geology). Comparing both acoustic methods, the AUV’s Doppler navigation was also much smoother than what could be achieved with the ROVs’ navigation systems in these complex environmental settings. With the ROV flying in close proximity to the wall, Doppler lock could not be reliably achieved and the noise associated with USBL systems in deeper waters required the use of pre- and post-processing smoothing filters. Experimenting with changes in the MBES configuration on hovering type AUVs will likely provide an additional set of tools to increase the efficiency of very high resolution vertical mapping.

### Future Directions

The manual classification employed in this study was useful to demonstrate the possibilities associated with SfM coloured point clouds; however, it remained subjective and time consuming. Automated classification of point clouds has received much attention with respect to LIDAR/laser scanning in complex natural settings^44–47^, but extension to very high-resolution photogrammetry-derived point clouds has also been successful^48^. Advances in seafloor optical imaging systems, such as the wide-range field-of-view camera system employed by GEOMAR that allows images to be acquired from a higher altitude (up to 9 m) and using a deep-sea AUV, will ensure that much greater coverage can be obtained rapidly^26^. Recent improvements on colour correction approaches for underwater 3D reconstructions, which make use of the 3D structural information, will also greatly improve model quality and may allow for colours to be more reliably utilized in automated classifications^49^. Developments associated with laser scanners on underwater vehicles will soon provide even more tools to complement those presented in this study^50, 51^.

### Conclusion

Deep-sea habitats with vertical components can afford protection to sometimes rich and diverse communities against activities such as trawling, but lost fishing gear, litter and climate change continue to pose imminent threats. The techniques presented in this paper allow greater resolutions and quantitative measures to be obtained, showing great promise in increasing our ability to map and monitor vertical structures, now increasingly likely to also include offshore infrastructure. Although likely frequent along the continental slope, complex wall habitats have so far not been examined in great detail, but the very high resolutions now achievable will enable us to better understand the additional fine-scale niches provided by such environments and their potential role in maintaining regional biodiversity.

## Electronic supplementary material

Supplementary Figure Captions

Supplementary Information S1

Supplementary Information S2 (3D pdf)

Supplementary Information S3 (3D pdf)

Supplementary Information S4

Supplementary Information S5

Supplementary Information S6 (3D pdf)
