# Supplementary material for: New approaches to high-resolution mapping of marine vertical structures
Source: Sci Rep. 2017 Aug 21;7:9005. doi: 10.1038/s41598-017-09382-z (PMC5567197; doi:10.1038/s41598-017-09382-z)
Supplement: Supplementary file 1 — Supplementary Figure Captions [file 41598_2017_9382_MOESM1_ESM.pdf]

New approaches to high-resolution mapping of marine vertical structures

Katleen Robert, Veerle A. I. Huvenne, Aggeliki Georgiopoulou, Daniel O. B. Jones, Leigh Marsh, Gareth D.O. Carter, and Leo Chaumillon

SUPPLEMENTARY INFORMATION

Supplementary Information S1: Fly through video showing the difference in resolution achievable with a sideways-looking AUV multibeam echosounder (MBES) (right) as compared to the ship-board MBES (left). Results for the Whittard Canyon Acesta Wall (depth range: 500-900m) are shown on top while the bottom shows the Whittard Canyon Coral Wall (depth range: 1,100-1,600m).

Supplementary Information S2: 3D model of the bathymetry obtained using a sideways-looking AUV multibeam echosounder for the Whittard Canyon Coral Wall (resolution: 5m).

Supplementary Information S3: 3D model of the bathymetry obtained using a sideways-looking AUV multibeam echosounder for the Whittard Canyon Acesta Wall (resolution: 0.5m)

Supplementary Information S4: Detailed description of the photogrammetry approach

Supplementary Information S5: Video animation showing a video frame slowly fading to reveal the results of the 3D photogrammetry reconstruction for a small section (~6m in length) of the Rockall Escarpment.

Supplementary Information S6: 3D model of the photogrammetry reconstruction for a small section (~6m in length) of the Rockall Escarpment.

Please note, not all pdf viewing software can display 3D models and the option to trust 3D content must be enabled in Adobe Reader.
