# Supplementary material for: New approaches to high-resolution mapping of marine vertical structures
Source: Sci Rep. 2017 Aug 21;7:9005. doi: 10.1038/s41598-017-09382-z (PMC5567197; doi:10.1038/s41598-017-09382-z)
Supplement: Supplementary file 5 — Supplementary Information S4 [file 41598_2017_9382_MOESM5_ESM.pdf]

# New approaches to high-resolution mapping of marine vertical structures

Katleen Robert, Veerle A. I. Huvenne, Aggeliki Georgiopoulou, Daniel O. B. Jones, Leigh Marsh, Gareth D.O. Carter, and Leo Chaumillon

**Supplementary Information 4 - Photogrammetry detailed approach**

1) Frames were extracted from the ROV video (using the freely available software Blender), and 1 frame for every three seconds of video (allowing for at least 60% overlap) was loaded into Agisoft photoscan (v1.2.4).

2) The USBL coordinates (UTM Zone 28N Rockall Escarpment and UTM Zone 29N Whittard Canyon) and attitude values (pitch, roll, heading) associated with each frame were loaded into the software from a text file. To account for the forward facing setup of the camera on the ROV (as opposed to the assumed downward facing camera used in aerial photography), a pitch offset of 90° was added.

3) If any part of the ROV's frame was visible, it was masked. The images were then aligned using the high accuracy setting and features were constrained by mask. This is the step when the software finds matches between images using structure invariant features, reconstructs the position of the cameras and produces a sparse point cloud. For faster processing, the feature matching search was constrained using the image reference location, whereby only images located nearby one another were compared.

4) A mesh was built from the sparse point cloud and multiple scale bars were created using the two lasers visible in the imagery (separated by 10cm). The camera parameters were optimized and an estimate of the error between the known laser distance and the reconstructed model was obtained. The estimated camera and marker positions were exported as text files while a model of the camera position was exported as a bundler file (.out) to be read in other 3D visualizing softwares.

5) A dense point cloud was created with high quality and mild depth filtering settings to remove outlier points. The dense point cloud was cleaned and exported as a xyz text file with both colour and normal information.

6) A mesh was created from the dense point cloud, the arbitrary surface type was selected, and the highest number of polygons was used (memory dependent). The model was textured using the generic mode and three texture files of size 4,096 x 4,096 pixels were created (Figure 3). The model was exported as a .obj file.
